# Profiles of Geriatric Syndromes and Resources in Older Patients with Atrial Fibrillation

**DOI:** 10.3390/jcm13144009

**Published:** 2024-07-09

**Authors:** Joshua Verleysdonk, Nicolas Noetzel, Ingrid Becker, Lena Pickert, Thomas Benzing, Roman Pfister, Maria Cristina Polidori, Anna Maria Affeldt

**Affiliations:** 1 Department II of Internal Medicine and Center for Molecular Medicine Cologne, Faculty of Medicine and University Hospital Cologne, University of Cologne, 50937 Cologne, Germanylena.pickert@uk-koeln.de (L.P.); thomas.benzing@uk-koeln.de (T.B.); anna.meyer@uk-koeln.de (A.M.A.); 2 Department of Oral and Maxillofacial Plastic Surgery, Evangelic Johanniter Hospital Bethesda Mönchengladbach, 41061 Mönchengladbach, Germany; nicolas.noetzel@mg.johanniter-kliniken.de; 3 Institute of Medical Statistics and Computational Biology, University Hospital of Cologne, 50937 Cologne, Germany; ingrid.becker@uni-koeln.de; 4 Cellular Stress Responses in Aging-Associated Diseases (CECAD), Faculty of Medicine and University Hospital Cologne, University of Cologne, 50937 Cologne, Germany; 5 Department III of Internal Medicine, Department of Cardiology, Faculty of Medicine and University Hospital Cologne, University of Cologne, 50937 Cologne, Germany; roman.pfister@uk-koeln.de

**Keywords:** Multidimensional Prognostic Index (MPI), frailty, geriatric resources (GRs), geriatric syndromes (GSs), atrial fibrillation (AF)

## Abstract

**Objective:** Older patients with nonvalvular atrial fibrillation (AF) are at high risk for frailty and geriatric syndromes (GSs), which modulate their individual prognosis and are therefore relevant for further management. Because few studies have evaluated the geriatric profile of older AF patients, this secondary analysis aims to further characterize the patterns of GSs and geriatric resources (GRs) in AF patients and their association with anticoagulation use. **Methods:** Data from 362 hospitalized patients aged 65 years and older with AF (n = 181, 77.8 ± 5.8 years, 38% female) and without AF (non-AF [NAF]; n = 181, 77.5 ± 5.9 years, 40% female) admitted to an internal medicine and nephrology ward of a large university hospital in Germany were included. All patients underwent usual care plus a comprehensive geriatric assessment (CGA) including calculation of the Multidimensional Prognostic Index (MPI) and collection of 17 GSs and 10 GRs. Patients were followed up by telephone 6 and 12 months after discharge to collect data on their health status. **Results:** The mean MPI score of 0.47 indicated an average risk of poor outcome, and patients with AF had a significantly higher MPI than those without AF (*p* = 0.040). After adjustment for chronological age, biological sex, Cumulative Illness Rating Scale (CIRS) for relevant chronic diagnoses and MPI as a proxy for biological age, AF patients had significantly more mnestic resources (63.5% vs. 33.1%, *p* < 0.001), a tendency for less age-appropriate living conditions (56.4% vs. 72.9%, *p* = 0.051) and more sensory impairment (78.5% vs. 52.5%, *p* < 0.001) than NAF patients. They also had a higher number of GSs (*p* = 0.046). AF patients on oral anticoagulants (OACs, n = 91) had less age-appropriate living conditions (48.4% vs. 64.4%, *p* < 0.05) and mnestic resources (36.3% vs. 54.4%, *p* < 0.01), but more emotional resources (80.2% vs. 65.6%, *p* < 0.05) and chronic pain (56% vs. 40%, *p* < 0.05) than patients without OACs (n = 90). Overall, mortality at 1 year was increased in patients with a higher MPI (*p* < 0.009, adjusted for age, sex and CIRS), with a diagnosis of AF (*p* = 0.007, adjusted for age, sex, CIRS and MPI), with of male sex (*p* = 0.008, adjusted for age, CIRS and MPI) and those with AF and treated with hemodialysis (*p* = 0.022, compared to AF patients without dialysis treatment). **Conclusions:** Patients with AF and patients with AF and OACs show differences in their multidimensional frailty degree as well as GR and GS profiles compared to patients without AF or with AF not treated with OACs. Mortality after 1 year is increased in AF patients with a higher MPI and dialysis, independently from OAC use and overall burden of chronic disease as assessed per CIRS. GRs and GSs, especially age-appropriate living conditions, emotional resources, sensory impairment and chronic pain, can be considered as factors that may modify the individual impact of frailty, underscoring the relevance of these parameters in the management of older patients.

## 1. Introduction

Atrial fibrillation (AF) is a common diagnosis with an incidence of 3.7%–4.2% in patients aged 60–70 years, and of 10–17% in patients aged 80 years and older [1]. Because age is an independent risk factor for AF [2], the incidence of AF is estimated to reach 14–17 million in Europe by 2030 [1]. Patients with untreated AF are known to have a fivefold increased risk of stroke compared to the general population [3], which calls for robust evidence-based detection and treatment. The decision to treat AF with anticoagulants is based on clinical scores (i.e., the CHA2DS2-VASc score to determine the stroke risk and the HASBLED score to be able to assess the risk of bleeding) [4]. Studies have shown that patients with a higher overall frailty are treated less frequently with oral anticoagulants (OACs) [5]. In the recently published EUROSAF-Study, the main findings show that anticoagulants, whether vitamin K antagonists (VKAs) or direct oral anticoagulants (DOACs), are more often prescribed to patients with a low-risk value in the Multidimensional Prognostic Index (MPI) compared to those with higher mean MPI values [6].

Less is known about the association with noncardiovascular events, particularly geriatric syndromes (GSs) and conditions such as dementia, depression, instability and incontinence [7]. GSs present as a concept of disability and a decline of function, which can hardly be traced to a single disease, but is rather a sum of effects caused by different conditions in addition to physiological aging processes [8]. A recent review showed that GSs have a major impact on noncardiovascular outcomes and that healthcare professionals need to consider these complex dynamics when managing AF in older adults [7]. The number of GSs has been shown to increase with the degree of comorbidity in patients with AF [9]. It has been hypothesized that the high prevalence of GSs may explain the lower than expected use of anticoagulants in older adults [10].

In addition to GSs, which are associated with negative outcomes [11], there are also geriatric resources (GR), mainly psychological factors such as motivational resources or emotional resources, which are associated with positive health outcomes and protect older patients in this regard [11]. In the literature, psychological factors have been suggested to influence the onset, progression, severity and outcomes of AF, but their role is unclear and mainly focused on anxiety and depression [12].

GSs and GRs can be assessed by a targeted comprehensive geriatric assessment (CGA). The Multidimensional Prognostic Index (MPI) as a prognostic CGA is calculated by dividing the relative score of its components by their number, resulting in an index between 0 and 1, with an increasing value being positively correlated with frailty. The MPI has been shown to be associated with mortality risk [13] and other health outcomes, such as length of hospital stay or grade of care [14] in hospitalized older patients. It is also associated with the occurrence of GSs and GRs [11]. Divided into three categories (MPI-1 ≤ 0.33 (low mortality risk), MPI-2 0.34–0.66 (intermediate risk) and MPI-3 > 0.66 (high risk)), it provides an appropriate framework to capture the burden of frailty.

The aim of this secondary analysis of the Cologne patients of the EUROSAF (EURopean study of Older Subjects with Atrial Fibrillation [6]) study was to investigate the prevalence and impact of GSs and GRs in older hospitalized patients with AF compared with non-AF (NAF) patients, and their association with anticoagulation use, paving the way for targeted, routine screening and treatment of these prognostically relevant conditions.

## 2. Methods

### 2.1. Study Population

This analysis includes data from 181 AF patients enrolled at the Department of Internal Medicine, University Hospital of Cologne, Germany as part of the EUROSAF [6] study, a multicenter study of a total of 2012 AF patients designed to evaluate the risk–benefit ratio in older patients with respect to mortality, thromboembolic events and bleeding events. Participants were at least 65 years of age and had previously been diagnosed with nonvalvular AF, were hospitalized at the time of recruitment (the initial assessment was performed between day 2 and 4 of the stay), were able to communicate in the German language and agreed to participate. Of the patients screened at the University Hospital of Cologne between 2016 and 2020, 185 met the inclusion criteria and signed the informed consent. Four cases with incomplete MPI data were subsequently excluded.

**Figure 1:**
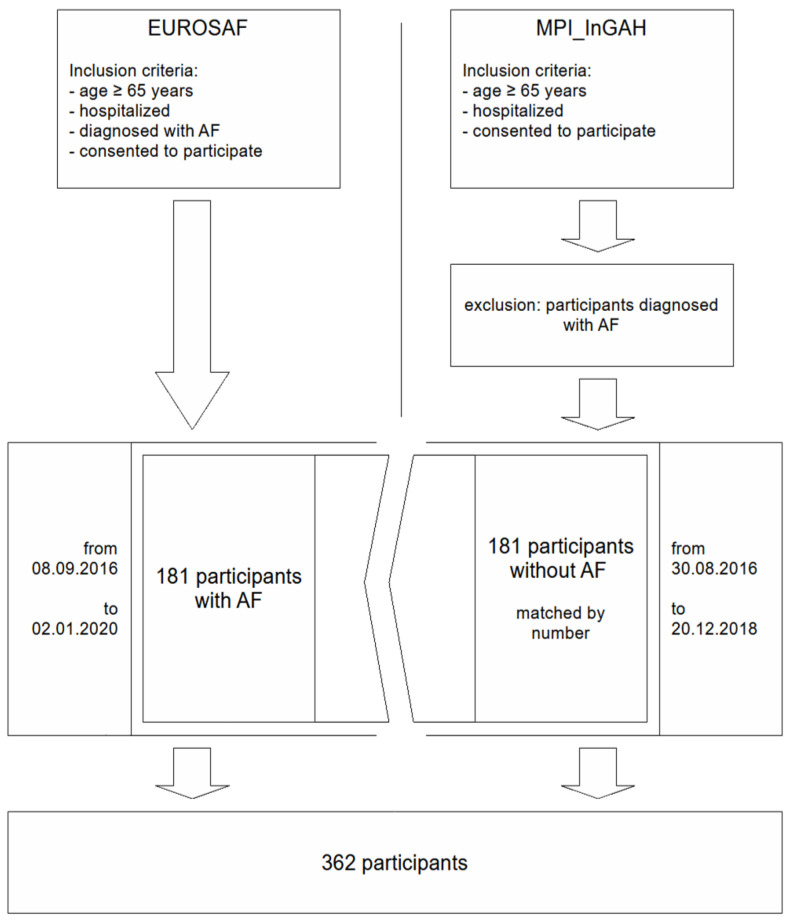
Flowchart of the study population. AF = atrial fibrillation.

In addition, 181 NAF patients from the same unit who participated in the prospective MPI_*InGAH*-study (Influence of Geriatric Assessment on Hospitalization) [11,14,15,16] were included in the data set. The matched selection of these patients was based on the date of participation (30 August 2016–20 December 2018), and participants with AF were excluded. The final dataset consisted of 362 cases. The mean age was 77.7 ± 5.8 years, and 39% were female (AF: *n* = 181, 77.8 ± 5.8 years, 38% female; NAF: *n* = 181, 77.5 ± 5.9 years, 40% female). The ward from which patients were recruited for both studies has a focus on general internal medicine, nephrology, hypertension and diabetology. The patients included in the present study are typical older multimorbid inpatients and had a wide range of diagnoses as well as a large variance in admission diagnosis. Common chronic diagnoses according to Cumulative Illness Rating Scale (CIRS) included renal disease (54.4%), metabolic disease (33.1%), blood or lymphatic vessel disease (21.5%) and respiratory or cardiac disease (each 21.3%) (Table 1). The most common reasons for admission to the ward and in the study population were renal failure (41.2%) and acute infection (21%) followed by cardiovascular diseases (6.9%). The patients included in this secondary analysis are shown in Figure 1. Except for the selection based on AF, no other inclusion/exclusion criteria as the ones stated had been made. Table 1 provides an overview of the demographical characteristics of the sample.

### 2.2. Assessments

After providing informed consent, participants were assessed using the CGA-based MPI [13], GSs and GRs [11] and medical history. The MPI included items to assess the Cumulative Illness Scale (CIRS) [17], Activities of Daily Living (ADL) [18], Instrumental Activities of Daily Living (IADL) [19], the Short Portable Mental Status Questionnaire (SPMSQ) [20], the Mini-Nutritional Assessment-Short Form (MNA-SF) [21], the Exton Smith Scale (ESS) [22], general living conditions and the number of medications that the patient was currently taking. The MPI was calculated, resulting in a score between 0 and 1. Depending on this score, the patients could be divided into three subgroups (MPI-1 (robust) 0–0.33, MPI-2 (prefrail) 0.34–0.66 and MPI-3 (frail) 0.67–1) according to their risk of death and risk of adverse health outcomes [13]. In a further step, the CIRS comorbidity index was analyzed separately in order to compare the overall burden of disease in terms of medical diagnoses between the groups. Since in the MPI calculation the CIRS is defined as “severe” with only 3 points, a direct comparison of the CIRS in the AF and NAF groups, which is greater than 3 in the vast majority of cases, provided a good opportunity to compare comorbidities. In addition, the subcategories of the admission score were analyzed to better compare the chronic disease characteristics of the groups.

For the analysis of patients with AF, the CHA2DS2-VASc and HASBLED scores were also collected. A total of 10 different GRs (i.e., physical, social, financial, spiritual, motivational, emotional, mnestic, competence-related, intellectual resources and age-appropriate living conditions) and 17 GSs (i.e., incontinence, instability, immobility, cognitive impairment, inanition, chronic pain, polypharmacy, irritability, sensory impairment, insomnia, irritable colon, iatrogenic disease, incoherence/delirium, impoverishment, isolation, fluid/electrolyte problems, dysphagia) were assessed as previously described [11]. If AF was diagnosed, the assessment included the anticoagulant agent that patients received on admission, if documented. All patients were followed up by telephone at 6 and 12 months for survival, rehospitalization, falls, medication and medical care.

### 2.3. Statistical Analysis

The study population (n = 362) was divided into the following two subgroups: AF and NAF patients. The variables age, sex, MPI, geriatric syndromes, geriatric resources, their number and whether the patient underwent regular hemodialysis were then tested in the two subgroups using the chi-squared test for nominal variables and the T-test or Mann–Whitney U-test for metric variables, depending if the variables were normally distributed. Effect size (Cohen) was calculated for significant differences concerning the individual MPI elements. To compare the burden of chronic diseases, we extracted the CIRS score of the CGA and compared it separately in both cohorts.

Furthermore, the relationship between GRs and GSs was described with a variable that was positive if the patient had relatively more GRs than GSs (in %). The results with significant *p*-values (*p* < 0.05) were then tested in a binary logistic regression adjusted for age, sex and MPI (Table 1). In order to assess the impact of GRs and GSs on the prescription of oral anticoagulants (OACs), the subgroup of AF patients (n = 181) was examined in the next step using the same statistical operations (Table 2). In addition, the use of OACs was summarized to further describe the study population (Table 3). In order to calculate the influence on mortality after 12 months in the AF group, the odds ratio was determined in addition to the statistical operations mentioned above (Table 4). Calculations were performed using SPSS (Statistical Package for Social Sciences, SPSS Inc., Chicago, IL, USA, version 26). Graphics were performed with GraphPad Prism Version 10.2.0 (392).

## 3. Results

### 3.1. Demographic Characteristics of the Study Sample

**Figure 2:**
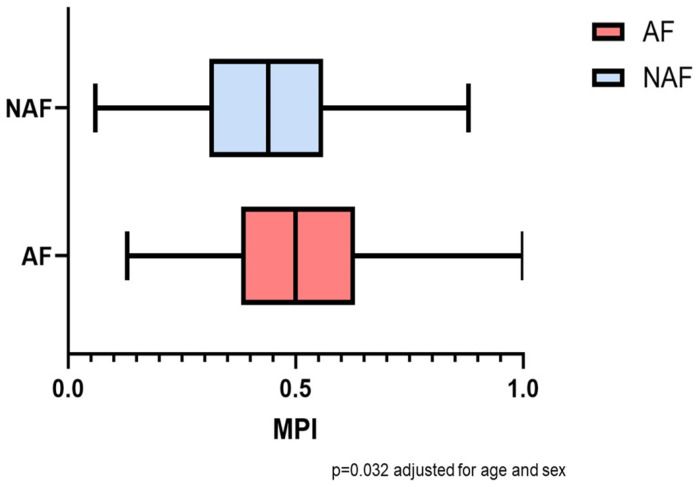
MPI distribution according to AF/NAF group. AF = atrial fibrillation; NAF = no atrial fibrillation; MPI = Multidimensional Prognostic Index.

Of the 362 patients included, the mean age was 77.7 years and 39% were female, with no significant difference in age or sex between the AF and NAF groups. The MPI showed a significant difference, with the mean index being higher in AF patients (*p* = 0.040, Figure 2), adjusted for age, sex and CIRS comorbidity index. Of the patients, 22.4% had MPI-1, 62.2% had MPI-2 and 15.5% had MPI-3, with no significant difference between the groups (Table 1). The mean CIRS score at discharge was 4.7 (SD 1.86) for the NAF group and 5.98 (SD 1.68) for the AF group, showing that AF patients had significantly more comorbidities (*p* < 0.001) than NAF patients, with a medium effect size (r = 0.317). With regard to CIRS subcategories at admission, a significant difference between groups was observed for severe diseases of the upper intestinal tract, albeit with a low incidence overall in both groups (3.3% AF group vs. 8.3% NAF group, *p* = 0.043), and for severe hypertension (4.4% AF group vs. 10.5% NAF group, *p* = 0.028). There was no significant difference in the prevalence of hemodialysis between the groups (*p* = 0.092). Patients with AF received more medications than patients without AF (*p* = 0.004), with weak effect size (r = 0.149). Of the remaining MPI subcategories, the SPMSQ was higher in patients with AF (*p* = 0.002) with weak effect size (r = 0.162). ADL, IADL, MNA-SF and ESS were not significantly different.

In this study of 181 participants with AF (mean age = 78 years (SD 5.8); mean CHA2DS2-VASc score = 4.67 (SD 1.51), mean HASBLED-score = 2.73 (SD 0.89)), the most common GSs were polypharmacy (82.9%), sensorial impairment (78.5%), instability (76.8%), insomnia (49.7%) and chronic pain (48.1%).

### 3.2. GRs and GSs According to AF/NAF Group

The mean number of GRs was 5.8 (SD 2), with no significant difference between the AF and NAF groups (*p* > 0.5), adjusted for age and sex. The mean occurrence of GSs was 5.66 (SD 2.5), where a significant difference could be shown with AF patients having significantly more GSs (*p* = 0.046).

Binary logistic regression showed a negative association between AF and age-appropriate living conditions (*p* < 0.05), indicating a higher proportion of age-appropriate living conditions in the NAF group. A positive association was found for mnestic resources (*p* < 0.001) and sensory impairment (*p* < 0.001, Table 1) with AF, adjusted for age, sex and MPI.

### 3.3. GRs and GSs in the AF Group According to OACs Use

Overall, 49.2% of the AF patients reported anticoagulant use, and the other 50.8% of patients were not on anticoagulant therapy on hospital admission. Regarding the prescription of OACs on admission, 32% of the patients were taking vitamin K antagonists (VKAs), 8.3% apixaban, 0.6% dabigatran, 1.7% edoxaban and 3.3% rivaroxaban (Table 2). Additionally, 36.4% were receiving antiplatelet therapy such as acetylsalicylic acid, often in combination with anticoagulants.

In the AF group, the difference between patients receiving OACs and those not receiving OACs was not significant with respect to the number of GRs and GSs (*p* > 0.5, Table 3). Of all AF patients discharged, 17.1% were treated with a DOAC, 18.8% with a VKA, 14.4% with a combination of OAC and antiplatelet drugs, and 49.7% received no anticoagulant treatment. The CIRS score indicated a comparable burden of severe chronic disease in both groups (*p* = 0.201). Binary logistic regression showed that the presence of anticoagulant treatment was negatively associated with regular hemodialysis (*p* < 0.01), spiritual resources (*p* < 0.01) and age-appropriate living conditions (*p* < 0.05). A positive association was found for emotional resources (*p* < 0.05) and chronic pain (*p* < 0.05), adjusted for age, sex and MPI, indicating that these patients were more likely to receive oral anticoagulant treatment (Table 3). In the study cohort, MPI did not show a significant association with treatment of AF with OACs (*p* > 0.1), adjusted for age and sex.

### 3.4. 1-Year Follow-Up Results

Overall, 72 (39.8%) AF patients died during the 1-year follow-up period (vs. 41 (22.7%) NAF patients, *p* = 0.007, adjusted for age, sex, CIRS and MPI). In the overall population (n = 362), mortality was significantly increased if the patients were male (*p* = 0.008), diagnosed with AF (*p* = 0.019) or had a higher MPI (*p* < 0.001), while dialysis and the CIRS comorbidity index had no significant impact on mortality (*p* = 0.099 and *p* = 0.721).

In the group of AF patients (n = 181), mortality was significantly increased if they underwent dialysis (*p* = 0.022) or had a higher MPI (*p* = 0.009, Table 4); if OACs were taken or not had no significant influence (*p* = 0.157). In AF patients, the number of GSs on admission was not associated with 1-year mortality after adjustment for age, sex, CIRS, dialysis and MPI (*p* = 0.578). The number of GRs on admission was also not associated with 1-year mortality after the same adjustment (*p* = 0.104). Mnestic GR and GS sensorial impairment had no effect on 1-year mortality in the AF vs. NAF group and in AF patients with or without OAC treatment, adjusted for age, sex and MPI (*p* > 0.050).

## 4. Discussion

The aim of this secondary analysis was to investigate the prevalence and impact of GSs and GRs in older hospitalized patients with AF compared with NAF patients, and their association with anticoagulation use. The analysis showed that the study subgroups were largely comparable in terms of demographics and disease burden (Table 1). The other MPI subcategories (beyond CIRS) showed partially significant differences between AF and NAF patients, although the effect size was small (Table 1). The recruitment setting was a specialized nephrology unit within general internal medicine. As such, patients typically present on this ward with a wide variety of acute and chronic diseases as well as an acute relapse of chronic illness [23,24]. We showed that the MPI was significantly higher in patients with AF, suggesting accelerated aging. In this context, it is noteworthy that while the CIRS score significantly differed between the AF and NAF groups, the MPI at discharge was significantly associated with a higher mortality at 12 months. Patients with AF died significantly more often during the year compared to patients with NAF, and the same applies to AF patients with dialysis and a higher MPI, independently from OAC treatment. The MPI biological age has been assessed in patients with heart disease [25] and previously in patients with AF [26] and has generally shown good predictive values. In a review, Villani et al. found that the prevalence of frailty can be four times higher in patients with AF and is an important prognostic indicator [27].

The decision to treat an older patient with anticoagulant therapy should be based on an assessment of the bleeding risk [28]. However, it also makes sense to assess the risk of falls—and the home environment is an important factor, because stairs, uneven showers or high cupboards can significantly increase the risk of falls and thus the risk of bleeding in everyday life. The decision in favor or against treatment is made considerably more difficult by dialysis therapy. Identifying frailty using multidimensional tools is essential to provide the best treatment for patients with AF [27,29]. Notably, 50.8% of the patient sample in this study were not receiving anticoagulant therapy on admission. It is known that the Deficit Accumulation Model (DAM) [30], which is very common when considering frailty, is associated with less use of OACs [5], although it has been shown that even in frail patients, anticoagulant use is still the treatment of choice when indicated [31]. The specific setting of this study with patients hospitalized on a ward with focus on renal diseases lead to a high percentage of participants with dialysis. In this group, there is still no clear recommendation for anticoagulant therapy among experts. It was shown that patients with AF who were regularly dialyzed were more likely to die, regardless of whether they received OACs or not.

The MPI did not show a significant association with treatment of AF with OACs in this study cohort. This finding suggests that an objective assessment of frailty may not have played a role in the treatment decisions. Patients benefit from treatment with OACs regardless of their frailty status [31,32,33], although in practice, frail patients are less likely to be treated with these agents [6,29]. We were able to show that AF patients had a higher number of GSs than NAF patients. Furthermore, the number of GSs and GRs did not seem to be important in AF patients, but the type of underlying GSs and GRs did. We found patterns of GSs and GRs that were specific to AF patients compared to NAF patients. Patients diagnosed with AF were less likely to have age-appropriate living conditions, more mnestic and emotional resources and more likely to have sensory impairment compared to NAF patients. However, the number of GSs and GRs was not shown to be prognostic for 1-year mortality. The role of GR, such as mnestic resources (definition: having many positive memories or success in the past [7]), has been largely neglected in most studies. It has previously been hypothesized that psychological distress, from which patients may be somewhat protected by mnestic resources, influences AF [12]. Galli et al. have also described the role of life events as a possible influence on AF that deserves further attention; because life events play a key role in mnestic resources, this may also be a link [12]. In particular, the study of personality traits in AF is in its infancy, but knowledge of personality traits that may predispose to AF may be useful from a prevention perspective [12].

In addition, patients with AF were significantly more likely to have sensorial impairment. According to the literature, there is some evidence that symptoms of sensory dysfunction led to the diagnosis of AF [34]. There is also evidence of an association between hearing impairment and AF, with significant associations between hearing impairment, hearing aid use and tinnitus with the risk of incident AF [35]. For visual impairment, one study showed an association between AF and visual impairment, but this appeared to be independently influenced by stroke severity [36]; another study found that type 2 diabetes mellitus in patients with incident AF was independently associated with increased severe visual impairment [37]. Whether AF is associated with more severe sensory impairment remains unknown, and more research is needed in this area.

AF patients were more likely to live in age-appropriate conditions if they were not treated with OACs. Previous literature has shown that poor living conditions, such as housing insecurity, are strongly associated with chronic diseases, including cardiovascular disease, longer hospital stays, more frequent emergency department visits and poorer access to preventive and primary care [38,39]. An article by Sims et al. showed that the increased prevalence of barriers to cardiovascular disease care among people facing housing problems further exacerbates health inequalities [40]. Thus, addressing the contribution of housing as an important social determinant of cardiovascular health requires a nuanced understanding of housing insecurity and a multifaceted approach that addresses each of these areas in an effort to reduce associated health inequalities [41].

This study also showed that the presence of anticoagulant treatment was negatively associated with spiritual resources (*p* < 0.01) and positively associated with emotional resources (*p* < 0.05) and chronic pain (*p* < 0.05). Because patients on anticoagulant treatment had fewer spiritual resources, it can be suggested that a relationship between spirituality and overall medication use (or compliance) may be the reason for this. Although previous studies have not found this to be the case in other heart disease patients [42], further studies are needed to describe the effect of spirituality on the management of chronic diseases such as AF. Another explanation may be that spiritual resources have been shown to be significantly associated with a higher MPI^7^, which indicates an overall higher risk of frailty and mortality in patients [13]. From an individual perspective, spiritual resources act as a coping strategy in stressful (i.e., critical) health-related situations [43]. This may indicate the conditions that physicians find when deciding not to treat AF patients with OACs. However, a causal relationship that leads physicians not to treat spiritual patients with OACs seems very unlikely.

The finding that emotional resources were associated with a higher use of OACs in this study cohort is consistent with a Danish nationwide cohort study showing that comorbid depression was associated with significantly lower use of OACs in patients with AF, raising the question of whether depressed patients receive sufficient support to manage this serious cardiac condition [44]. Because elevated symptoms of depression and, to a lesser extent, symptoms of anxiety are independently associated with all-cause mortality in outpatients with OACs, depressiveness should be considered a clinically significant condition that needs to be addressed in the management of anticoagulation patients [45].

Because patients on OACs had a significantly higher prevalence of chronic pain than GSs in this study cohort, this is consistent with previous findings showing that coexisting symptomatic AF may be associated with a higher pain sensitization in general [34] and which may provide an avenue for further investigation of the relationship between chronic pain and OAC prescription. Of the approved agents, only edoxaban and acetylsalicylic acid have documented headache and abdominal pain as possible adverse effects. In our study population, only a small percentage were receiving these agents, so an association with chronic pain seems unlikely. However, why chronic pain was more common in patients on OAC therapy in this cohort is not well explained in the existing literature and is certainly another factor that needs to be investigated.

### Limitations of This Study

This study was conducted with patients in a specific setting, a nephrology unit, where atrial fibrillation was a comorbidity, and therefore the number of participants may have been small compared to other internal medicine settings. Further research to investigate the association of GRs and GSs with common diagnoses in older patients is therefore warranted.

Because two different cohorts were analyzed for this study, only limited conclusive statements are possible despite comparable demographic data and disease burden. In addition, the retrospective nature of the investigation and the relatively small sample size do limit the generalizability of conclusions. However, the deep clinical characterization of study participants as far as comprehensive assessment, functions and geriatric complex conditions go allowed the spotlight of factors of high potential for improving clinical decision making. This is particularly important in light of the upcoming quadruplication of the oldest-old population.

Certainly, the most important limitation of OACs is the high rate of dialysis dependency of the patients analyzed, which, due to the recruitment setting, originated from a nephrology unit. Hemodialysis patients may therefore be overrepresented in this analysis. With a prevalence of up to 27%, AF is a common comorbidity in patients with end-stage renal disease receiving hemodialysis [46]. Current evidence on anticoagulation in hemodialysis patients with AF is based on largely conflicting observational data showing no clear benefit of vitamin K antagonists over no treatment for stroke prevention. Current guidelines do not provide specific consensus on whether or which OACs should be used in patients with end-stage renal disease (ESRD) on hemodialysis due to the increased risk of bleeding associated with anticoagulation treatment in these patients [47]. Because the follow-up could not be completed for some patients, the number of cases analyzed is relatively small. This may have had an influence on the strength of the measured effects, for example the lack of significant associations with mortality at follow-up for the MPI on admission.

## 5. Practical Conclusions

- Assessment of GRs and GSs provides insight into profiles and the impact of frailty in patients with AF.
- Although not the number of GRs and GSs does not appear to be important, mnestic resources and sensorial impairment are significantly more common in patients with AF and should be assessed.
- Emotional resourced and chronic pain are more common in patients receiving OACs, while spiritual resources are less common, but the relationship needs further investigation.

## Figures and Tables

**Table 1:** GRs and GSs according to AF/NAF group.

	Total N = 362 n (%)	AF N = 181 n (%)	NAF N = 181 n (%)	Univariate *p* Value *	*p* Value (Logistic Regression)
**Age**, mean (SD) **	77.65 (5.83)	77.78 (5.8)	77.53 (5.9)	0.686	0.551
**Female sex**	140 (38.7)	68 (37.6)	72 (39.8)	0.666	0.337
**Hemodialysis** after discharge	117 (32.3)	66 (36.5)	51 (28.2)	0.092	
**MPI admission**, mean (SD) **	0.49 (0.17)	0.51 (0.16)	0.46 (0.18)	0.032	0.021
**MPI discharge**, mean (SD) **	0.47 (0.16)	0.49 (0.15)	0.45 (0.17)	0.263	0.040
MPI subcategories (discharge), median (IQR) ***					
**CIRS comorbidity index (discharge), *mean* (SD)**	5.38 (1.87)	5.98 (1.68)	4.77 (1.86)	<0.001, r = 0.317	<0.001
**Activities of Daily Living**	5 (3)	5 (3)	5 (3)	0.237	
**Instrumental Activities of Daily Living**	5 (4)	5 (4)	5 (4)	0.438	
**Mini Nutritional Assessment Short Form**	9 (6)	8 (6)	9 (7)	0.052	
**Short Portable Mental Status Questionnaire**	1 (2)	1 (3)	1 (2)	0.002, r = 0.162	
**Exton Smith Scale**	16 (6)	16 (4)	16 (7)	0.949	
**Number of drugs, *mean* (SD)**	9.73 (3.979)	10.31 (3.72)	9.16 (4.15)	0.004, r = 0.149	
**Living conditions**	1 (2)	1 (2)	1 (2)	0.537	
***CIRS subcategories (admission), n (%)***					
**CIRS subcategory: psychiatric disease**	6 (1.7)	4 (2.2)	2 (1.1)	0.410	
**CIRS subcategory: metabolic disease**	120 (33.1)	56 (30.9)	64 (35.4)	0.372	
**CIRS subcategory: neurological disease**	34 (9.4)	12 (6.6)	22 (12.2)	0.072	
**CIRS subcategory: musculoskeletal disease**	50 (13.8)	24 (13.3)	26 (14.4)	0.761	
**CIRS subcategory: genitourinary disease**	46 (12.7)	24 (13.3)	22 (12.2)	0.752	
**CIRS subcategory: kidney disease**	197 (54.4)	103 (56.9)	94 (51.9)	0.342	
**CIRS subcategory: liver disease**	10 (2.8)	4 (2.2)	6 (3.3)	0.521	
**CIRS subcategory: disease of lower ** **gastrointestinal tract**	20 (5.5)	11 (6.1)	9 (5.0)	0.645	
**CIRS subcategory: disease of upper** **gastrointestinal tract**	21 (5.8)	6 (3.3)	15 (8.3)	0.043	
**CIRS subcategory: disease of eyes, ears, nose, larynx, pharynx**	13 (3.6)	6 (3.3)	7 (3.9)	0.778	
**CIRS subcategory: respiratory disease**	77 (21.3)	35 (19.3)	42 (23.2)	0.369	
**CIRS subcategory: vascular, hematological, lymphatic disease**	78 (21.5)	37 (20.4)	41 (22.7)	0.609	
**CIRS subcategory: hypertension**	27 (7.5)	8 (4.4)	19 (10.5)	0.028	
**CIRS subcategory: heart disease**	77 (21.3)	37 (20.4)	40 (22.1)	0.700	
***GRs and GSs***					
Number of **GRs**, mean (SD) **	5.8 (2)	5.97 (2.1)	6.64 (1.9)	0.117	
**Physical resources**	153 (42.3)	68 (37.6)	85 (47)	0.070	0.308
**Age-appropriate living conditions**	234 (64.6)	102 (56.4)	132 (72.9)	<0.001	0.051
**Social resources**	313 (86.5)	152 (84)	161 (89)	0.167	
**Financial resources**	213 (58.8)	106 (58.6)	107 (59.1)	0.915	
**Spiritual resources**	152 (42)	82 (45.3)	70 (38.7)	0.201	
**Motivational resources**	224 (61.9)	124 (68.5)	100 (55.2)	0.009	0.014
**Emotional resources**	255 (70.4)	132 (72.9)	123 (68)	0.300	
**Mnestic resources**	175 (48.3)	115 (63.5)	60 (33.1)	<0.001	<0.001
**Competence-related resource**	171 (47.2)	89 (49.2)	82 (45.3)	0.461	
**Intellectual resources**	195 (53.9)	109 (60.2)	86 (47.5)	0.015	0.316
Number of **GSs**, mean (SD) **	5.66 (2.5)	6.24 (2.4)	5.07 (2.6)	0.746	0.046
**Incontinence**	146 (40.3)	70 (38.7)	76 (42)	0.520	
**Instability**	242 (66.9)	139 (76.8)	103 (56.9)	<0.001	0.220
**Immobility**	142 (39.2)	77 (42.5)	65 (35.9)	0.196	
**Cognitive Impairment**	35 (9.7)	17 (9.4)	18 (9.9)	0.859	
**Inanition**	136 (37.6)	74 (40.9)	62 (34.3)	0.193	
**Chronic Pain**	161 (44.5)	87 (48.1)	74 (40.9)	0.169	
**Polypharmacy**	297 (82)	150 (82.9)	147 (81.2)	0.681	
**Irritability/Depression**	62 (17.1)	32 (17.7)	30 (16.6)	0.780	
**Sensorial impairment**	237 (65.5)	142 (78.5)	95 (52.5)	<0.001	0.003
**Insomnia**	165 (45.6)	90 (49.7)	75 (41.4)	0.113	
**Irritable colon**	142 (39.2)	71 (39.2)	71 (39.2)	1.000	
**Iatrogenic disease**	38 (10.5)	23 (12.7)	15 (8.3)	0.170	
**Incoherence/Delirium**	14 (3.9)	9 (5)	5 (2.8)	0.276	
**Impoverishment**	22 (6.1)	13 (7.2)	9 (5)	0.379	
**Isolation**	23 (6.4)	11 (6.1)	12 (6.6)	0.829	
**Fluid/Electrolyte problems**	114 (31.5)	69 (38.1)	45 (24.9)	0.007	0.218
**Swallowing disorder**	59 (16.3)	36 (19.9)	23 (12.7)	0.064	0.531
**GRs (%) > GSs (%)**	294 (81.2)	148 (81.8)	146 (80.7)	0.788	

* *p* values significant at 5% level, logistic regression performed if significant. Chi-square test for nominal variables, ** *t*-test (for normally distributed) and *** Mann–Whitney U-Test for metric variables, Table notes: r = effect size, AF = atrial fibrillation, NAF = no atrial fibrillation, CIRS = Cumulative Illness Rating Scale, SD = standard deviation, IQR = interquartile range, MPI = Multidimensional Prognostic Index.

**Table 2:** Anticoagulant and antiplatelet prescription components on admission.

	OACs Total N = 89 N (%)
**VKAs prescription** *(N = 181)*	58 (32)
**DOACs prescription** *(N = 181)*	31 (17.1)
Apixaban	15 (8.3)
Dabigatran	1 (0.6.)
Edoxaban	3 (1.7)
Rivaroxaban	6 (3.3)
Component not specified	6 (3.3)
**Antiplatelet therapy**	66 (36.4)

Table notes: VKAs = vitamin K antagonists, OACs = oral anticoagulants, DOACs = direct oral anticoagulants.

**Table 3:** Study population of subjects diagnosed with AF.

	Total N = 181 n (%)	OACs N = 91 n (%)	No OACs N = 90 n (%)	Univariate *p* Value *	*p* Value (Logistic Regression)
**Age**, mean (SD) **	77.78 (5.8)	77.19 (5.6)	78.38 (5.9)	0.167	
**Female**	68 (37.6)	35 (38.5)	33 (36.7)	0.803	
**CIRS comorbidity index (discharge), *mean* (SD)**	5.98 (1.68)	5.82 (1.91)	6.14 (1.40)	0.201	
**Hemodialysis** after discharge	66 (36.5)	24 (26.4)	42 (46.7)	0.005	0.003
**MPI admission**, mean (SD) **	0.52 (0.16)	0.50 (0.16)	0.53 (0.16)	0.179	
**MPI discharge**, mean (SD) **	0.49 (0.15)	0.48 (0.15)	0.51 (0.15)	0.164	
**CHA2DS2-VASc**, mean (SD) **	4.67 (1.5)	4.74 (1.4)	4.60 (1.6)	0.546	
**HAS-BLED**, mean (SD) **	2.73 (0.9)	2.66 (0.9)	2.81 (0.9)	0.250	
Number of **GRs**, mean (SD) **	5.97 (2.1)	5.92 (1.96)	6.01 (2.2)	0.777	
**Physical resources**	68 (37.6)	32 (35.2)	36 (40)	0.502	
**Age-appropriate living conditions**	102 (56.4)	44 (48.4)	58 (64.4)	0.029	0.021
**Social resources**	152 (84)	74 (81.3)	78 (86.7)	0.327	
**Financial resources**	106 (58.6)	57 (62.6)	49 (54.4)	0.263	
**Spiritual resources**	82 (45.3)	33 (36.3)	49 (54.4)	0.014	0.002
**Motivational resources**	124 (68.5)	67 (73.6)	57 (63.3)	0.136	
**Emotional resources**	132 (72.9)	73 (80.2)	59 (65.6)	0.026	0.010
**Mnestic resources**	115 (63.5)	62 (68.1)	53 (58.9)	0.196	
**Competence-related resource**	89 (49.2)	46 (50.5)	43 (47.8)	0.709	
**Intellectual resources**	109 (60.2)	53 (58.2)	56 (62.2)	0.584	
Number of **GSs**, mean (SD) **	6.24 (2.4)	6.18 (2.46)	6.31 (2.25)	0.700	
**Incontinence**	70 (38.7)	32 (35.2)	38 (42.2)	0.330	
**Instability**	139 (76.8)	73 (80.2)	66 (73.3)	0.272	
**Immobility**	77 (42.5)	32 (35.2)	45 (50)	0.044	0.246
**Cognitive Impairment**	17 (9.4)	7 (7.7)	10 (11.1)	0.431	
**Inanition**	74 (40.9)	37 (40.7)	37 (41.1)	0.951	
**Chronic Pain**	87 (48.1)	51 (56)	36 (40)	0.031	0.040
**Polypharmacy**	150 (82.9)	74 (81.3)	76 (84.4)	0.577	
**Irritability/Depression**	32 (17.7)	14 (15.4)	18 (20)	0.416	
**Sensorial impairment**	142 (78.5)	69 (75.8)	73 (81.1)	0.387	
**Insomnia**	90 (49.7)	48 (52.7)	42 (46.7)	0.413	
**Irritable colon**	71 (39.2)	35 (38.5)	36 (40)	0.832	
**Iatrogenic disease**	23 (12.7)	12 (13.2)	11 (12.2)	0.846	
**Incoherence/Delirium**	9 (5)	5 (5.5)	4 (4.4)	0.745	
**Impoverishment**	13 (7.2)	6 (6.6)	7 (7.8)	0.758	
**Isolation**	11 (6.1)	6 (6.6)	5 (5.6)	0.770	
**Fluid/Electrolyte problems**	69 (38.1)	36 (39.6)	33 (36.7)	0.689	
**Swallowing disorder**	36 (19.9)	19 (20.9)	17 (18.9)	0.737	
**GRs (%) > GSs (%)**	148 (81.8)	77 (84.6)	71 (78.9)	0.318	

* *p* values significant at 5% level, logistic regression performed if significant. Chi-square test for nominal variables, ** *t*-test for metric variables; Table notes: AF = atrial fibrillation, NAF = no atrial, CIRS = Cumulative Illness Rating Scale fibrillation, OACs = oral anticoagulants, SD = standard deviation, MPI = Multidimensional Prognostic Index.

**Table 4:** Survival after 12 months in the AF group.

	Total N = 154 n (%)	Survived N = 82 n (%)	Not Survived N = 72 n (%)	Univariate *p* Value *	*p* Value (Logistic Regression)	Odds Ratio (Lower/Upper Value of 95% Confidence Interval)
**Age**, mean (SD) **	77.45 (5.9)	76.93 (5.8)	78.04 (6.0)	0.244	0.446	0.977 (0.922/1.036)
**Female**	60 (39)	37 (45.1)	23 (31.9)	0.094	0.084	1.848 (0.920/3.711)
**CIRS at discharge,** mean (SD) **	6.06 (1.67)	5.84 (1.81)	6.32 (1.47)	0.077		
**Hemodialysis** after discharge	60 (39)	23 (28)	37 (51.4)	0.003	0.022	0.440 (0.218/0.886)
**MPI admission**, mean (SD) **	0.51 (0.16)	0.48 (0.15)	0.55 (0.16)	0.005	0.814	0.579 (0.006/54.578)
**MPI discharge**, mean (SD) **	0.49 (0.15)	0.45 (0.12)	0.53 (0.17)	0.001	0.009	0.040 (0.004/0.448)
**OAC therapy**	80 (51.9))	49 (59.8)	31 (43.1)	0.038	0.157	1.649 (0.824/3.300)
Number of **GRs**, mean (SD) **	5.06 (2.1)	6.32 (2.1)	5.78 (2.0)	0.104		
Number of **GSs**, mean (SD) **	6.27 (2.4)	6.17 (2.33)	6.39 (2.50)	0.578		
**GRs (%) > GSs (%)**	127 (82.5)	70 (85.4)	57 (79.2)	0.313		

* *p* values significant at 5% level, logistic regression performed if significant. Chi-square test for nominal variables, ** *t*-test for metric variables; Table notes: AF = atrial fibrillation, NAF = no atrial, CIRS = Cumulative Illness Rating Scale fibrillation, OACs = oral anticoagulants, SD = standard deviation, MPI = Multidimensional Prognostic Index.

## Data Availability

The data are available upon reasonable request to the corresponding author.

## References

[1] Zoni-Berisso M., Lercari F., Carazza T., Domenicucci S. (2014). Epidemiology of atrial fibrillation: European perspective. Clin. Epidemiol..

[2] Benjamin E.J., Levy D., Vaziri S.M., D’Agostino R.B., Belanger A.J., Wolf P.A. (1994). Independent Risk Factors for Atrial Fibrillation in a Population-Based Cohort: The Framingham Heart Study. JAMA.

[3] Migdady I., Russman A., Buletko A.B. (2021). Atrial Fibrillation and Ischemic Stroke: A Clinical Review. Semin. Neurol..

[4] Eckardt L., Häusler K.G., Ravens U., Borggrefe M., Kirchhof P. (2016). ESC-Leitlinien zum Vorhofflimmern 2016. Herz.

[5] Presta R., Brunetti E., Polidori M.C., Bo M. (2022). Impact of frailty models on the prescription of oral anticoagulants and on the incidence of stroke, bleeding, and mortality in older patients with atrial fibrillation: A systematic review. Ageing Res. Rev..

[6] Pilotto A., Veronese N., Polidori M.C., Strandberg T., Topinkova E., Cruz-Jentoft A.J., Custodero C., Maggi S., On Behalf of the EUROSAF Study Investigators (2022). The role of prognostic stratification on prescription of anticoagulants in older patients with atrial fibrillation: A multicenter, observational, prospective European study (EUROSAF). Ann. Med..

[7] Alagiakrishnan K., Banach M., Mah D., Ahmed A., Aronow W.S. (2019). Role of Geriatric Syndromes in the Management of Atrial Fibrillation in Older Adults: A Narrative Review. J. Am. Med. Dir. Assoc..

[8] Inouye S.K., Studenski S., Tinetti M.E., Kuchel G.A. (2007). Geriatric Syndromes: Clinical, Research, and Policy Implications of a Core Geriatric Concept. J. Am. Geriatr. Soc..

[9] Vetrano D.L., Foebel A.D., Marengoni A., Brandi V., Collamati A., Heckman G.A., Hirdes J., Bernabei R., Onder G. (2016). Chronic diseases and geriatric syndromes: The different weight of comorbidity. Eur. J. Intern. Med..

[10] Shah S.J., Fang M.C., Jeon S.Y., Gregorich S.E., Covinsky K.E. (2021). Geriatric Syndromes and Atrial Fibrillation: Prevalence and Association with Anticoagulant Use in a National Cohort of Older Americans. J. Am. Geriatr. Soc..

[11] Meyer A.M., Becker I., Siri G., Brinkkötter P.T., Benzing T., Pilotto A., Polidori M.C. (2020). The prognostic significance of geriatric syndromes and resources. Aging Clin. Exp. Res..

[12] Galli F., Borghi L., Carugo S., Cavicchioli M., Faioni E.M., Negroni M.S., Vegni E. (2017). Atrial fibrillation and psychological factors: A systematic review. PeerJ.

[13] Pilotto A., Ferrucci L., Franceschi M., D’Ambrosio L.P., Scarcelli C., Cascavilla L., Paris F., Placentino G., Seripa D., Dallapiccola B. (2008). Development and Validation of a Multidimensional Prognostic Index for One-Year Mortality from Comprehensive Geriatric Assessment in Hospitalized Older Patients. Rejuvenation Res..

[14] Meyer A.M., Becker I., Siri G., Brinkkötter P.T., Benzing T., Pilotto A., Polidori M.C. (2019). New associations of the Multidimensional Prognostic Index. Z. Für Gerontol. Geriatr..

[15] Pickert L., Meyer A.M., Becker I., Heeß A., Noetzel N., Brinkkötter P., Pilotto A., Benzing T., Polidori M.C. (2021). Role of a multidimensional prognosis in-hospital monitoring for older patients with prolonged stay. Int. J. Clin. Pract..

[16] Heeß A., Meyer A.M., Becker I., Noetzel N., Verleysdonk J., Rarek M., Benzing T., Polidori M.C. (2022). The prognostic fingerprint of quality of life in older inpatients: Relationship to geriatric syndromes’ and resources’ profile. Z. Für Gerontol. Geriatr..

[17] Linn B.S., Linn M.W., Gurel L. (1968). Cumulative illness rating scale. J. Am. Geriatr. Soc..

[18] Katz S., Downs T.D., Cash H.R., Grotz R.C. (1970). Progress in Development of the Index of ADL. Gerontologist.

[19] Lawton M.P., Brody E.M. (1969). Assessment of older people: Self-maintaining and instrumental activities of daily living. Gerontologist.

[20] Pfeiffer E. (1975). A Short Portable Mental Status Questionnaire for the Assessment of Organic Brain Deficit in Elderly Patients†. J. Am. Geriatr. Soc..

[21] Sancarlo D., D’Onofrio G., Franceschi M., Scarcelli C., Niro V., Addante F., Copetti M., Ferrucci L., Fontana L., Pilotto A. (2011). Validation of a modified-multidimensional prognostic index (m-MPI) including the mini nutritional assessment short-form (MNA-SF) for the prediction of one-year mortality in hospitalized elderly patients. J. Nutr. Health Aging..

[22] Bliss M.R., McLaren R., Exton-Smith A.N. (1966). Mattresses for preventing pressure sores in geriatric patients. Mon. Bull. Minist. Health Public Health Lab. Serv..

[23] Kozieł M., Teutsch C., Halperin J.L., Rothman K.J., Diener H.-C., Ma C.-S., Marler S., Lu S., Gurusamy V.K., Huisman M.V. (2021). Atrial fibrillation comorbidities: Clinical characteristics antithrombotic treatment in GLORIA-AF. PLoS ONE.

[24] Heijman J., Linz D., Schotten U. (2021). Dynamics of Atrial Fibrillation Mechanisms and Comorbidities. Annu. Rev. Physiol..

[25] Pilotto A., Addante F., Franceschi M., Leandro G., Rengo G., D’Ambrosio P., Longo M.G., Rengo F., Pellegrini F., Dallapiccola B. (2010). Multidimensional Prognostic Index Based on a Comprehensive Geriatric Assessment Predicts Short-Term Mortality in Older Patients with Heart Failure. Circ. Heart Fail..

[26] Veronese N., Argusti A., Canepa E., Polidori M.C., Maggi S., Strandberg T., Pilotto A., EUROSAF Study Investigators (2018). Evaluating the effectiveness and risks of oral anticoagulant treatments in multimorbid frail older subjects with atrial fibrillation using the multidimensional prognostic index: The EURopean study of older subjects with atrial fibrillation—EUROSAF. Eur. Geriatr. Med..

[27] Villani E.R., Tummolo A.M., Palmer K., Gravina E.M., Vetrano D.L., Bernabei R., Onder G., Acampora N. (2018). Frailty and atrial fibrillation: A systematic review. Eur. J. Intern. Med..

[28] Cosmi B., Palareti G. (2009). Bleeding with anticoagulation therapy—Who is at risk, and how best to identify such patients. Thromb. Haemost..

[29] Polidori M.C., Alves M., Bahat G., Boureau A.S., Ozkok S., Pfister R., Pilotto A., Veronese N., Bo M., On behalf of the Special Interest Group “Cardiovascular Diseases” of the EuGMS (2021). Atrial fibrillation: A geriatric perspective on the 2020 ESC guidelines. Eur. Geriatr. Med..

[30] Mitnitski A.B., Mogilner A.J., Rockwood K. (2001). Accumulation of Deficits as a Proxy Measure of Aging. Sci. World J..

[31] Pilotto A., Veronese N., Polidori M.C., Strandberg T., Topinkova E., Cruz-Jentoft A., Custodero C., Maggi S. (2023). Frailty and anticoagulants in older subjects with atrial fibrillation: The EUROSAF study. Age Ageing.

[32] Zeng S., Zheng Y., Jiang J., Ma J., Zhu W., Cai X. (2022). Effectiveness and Safety of DOACs vs. Warfarin in Patients with Atrial Fibrillation and Frailty: A Systematic Review and Meta-Analysis. Front. Cardiovasc. Med..

[33] Søgaard M., Ording A.G., Skjøth F., Larsen T.B., Nielsen P.B. (2024). Effectiveness and safety of direct oral anticoagulation vs. warfarin in frail patients with atrial fibrillation. Eur. Heart J. Cardiovasc. Pharmacother..

[34] Jackson A., Frobert O., Larsen D.B., Arendt-Nielsen L., Björkenheim A. (2021). Patients with symptomatic permanent atrial fibrillation show quantitative signs of pain sensitisation. Open Heart..

[35] Hou Y., Yang H., Xu Y., Wang K., Fu Y., Lu Z. (2024). Hearing disorders, genetic predisposition, and risk of new-onset atrial fibrillation: A prospective cohort study in the UK biobank. Int. J. Cardiol..

[36] Rowe F.J., Hepworth L.R., Howard C., Cullen C., Sturgess B., Griffiths N., Lip G.Y.H. (2020). Stroke-Related Visual Impairment; is There an Association with Atrial Fibrillation?. J. Stroke Cerebrovasc. Dis..

[37] Kaewput W., Thongprayoon C., Rangsin R., Bathini T., Mao M.A., Cheungpasitporn W. (2021). Associations of new-onset atrial fibrillation and severe visual impairment in type 2 diabetes: A multicenter nationwide study. World J. Cardiol..

[38] Martin P., Liaw W., Bazemore A., Jetty A., Petterson S., Kushel M. (2019). Adults with Housing Insecurity Have Worse Access to Primary and Preventive Care. J. Am. Board. Fam. Med..

[39] Parekh T., Xue H., Cheskin L.J., Cuellar A.E. (2022). Food insecurity and housing instability as determinants of cardiovascular health outcomes: A systematic review. Nutr. Metab. Cardiovasc. Dis..

[40] Sims M., Kershaw K.N., Breathett K., Jackson E.A., Lewis L.M., Mujahid M.S., Suglia S.F., On Behalf of the American Heart Association Council on Epidemiology and Prevention and Council on Quality of Care and Outcomes Research (2020). Importance of Housing and Cardiovascular Health and Well-Being: A Scientific Statement from the American Heart Association. Circ. Cardiovasc. Qual. Outcomes.

[41] Latif A., Tran A.M., Ahsan M.J., Niu F., Walters R.W., Kim M.H. (2023). Relationship of health-related social needs and hospital readmissions in patients following a hospitalization for atrial fibrillation. Am. Heart J. Plus Cardiol. Res. Pract..

[42] Black G., Davis B.A., Mitchell B.N., Sanderson C. (2006). The Relationship Between Spirituality and Compliance in Patients with Heart Failure. Prog. Cardiovasc. Nurs..

[43] Badanta B., Rivilla-García E., Lucchetti G., De Diego-Cordero R. (2022). The influence of spirituality and religion on critical care nursing: An integrative review. Nurs. Crit. Care.

[44] Fenger-Grøn M., Vestergaard C.H., Frost L., Davydow D.S., Parner E.T., Christensen B., Ribe A.R. (2020). Depression and Uptake of Oral Anticoagulation Therapy in Patients with Atrial Fibrillation: A Danish Nationwide Cohort Study. Med. Care.

[45] Michal M., Prochaska J.H., Keller K., Göbel S., Coldewey M., Ullmann A., Schulz A., Lamparter H., Münzel T., Reiner I. (2015). Symptoms of depression and anxiety predict mortality in patients undergoing oral anticoagulation: Results from the thrombEVAL study program. Int. J. Cardiol..

[46] Königsbrügge O., Ay C. (2019). Atrial fibrillation in patients with end-stage renal disease on hemodialysis: Magnitude of the problem and new approach to oral anticoagulation. Res. Pract. Thromb. Haemost..

[47] Kyriakoulis I., Adamou A., Stamatiou I., Chlorogiannis D.D., Kardoutsos I., Koukousaki D., Ntaios G. (2024). Efficacy and safety of direct oral anticoagulants vs vitamin K antagonists in patients with atrial fibrillation and end-stage renal disease on hemodialysis: A systematic review and meta-analysis. Eur. J. Intern. Med..

